# Microstructural and mechanical characterization of Al/Cu interface in a bimetallic composite produced by compound casting

**DOI:** 10.1038/s41598-024-57849-7

**Published:** 2024-03-29

**Authors:** Shima Ahmadzadeh Salout, Seyed Mohammad Hossein Mirbagheri

**Affiliations:** Present Address: Department of Materials and Metallurgical Engineering, Amirkabir University of Technology, Tehran, 15875-4413 Iran

**Keywords:** Mechanical engineering, Engineering, Materials science

## Abstract

The bimetal set (Al/Cu) with Cu wire with 2.0, 2.5, and 3.0 mm diameters were cast at different casting temperatures and solidification times through the compound casting method. The microstructure of solid/liquid diffusion bonding at the Al/Cu interface was investigated, and the shear strength of the Al/Cu interface was measured by punch test. By characterizing the diffusion layer, the optimum parameters of the compound casting, including the casting temperature and the solidification soaking time, as well as the Cu wire diameter, were acquired. The intermetallic compounds (IMCs) such as CuAl_2_ were observed in the diffusion layer. The types of intermetallic phases and diffusion layer thickness affect the hardness and the shear strength. The result of casting at 680 °C and solidification soaking time of 15 s for 3 mm Cu wire, shows that IMCs increased the micro-hardness of the Al/Cu bimetal up to 328 HV at the Al/Cu interface. Also, increasing the solidification soaking time at a constant temperature resulted in a growth of the interface layer’s thickness, which exhibits a lamellar eutectic microstructure containing IMCs. Furthermore, this action caused an increase in the shear strength.

## Introduction

Bimetallic composites provide remarkable economic advantages by reducing material costs and the enhancement of functional capabilities. Combining individual metals’ behavior can be effectively attained using bimetallic composites (bimetals)^1,2^. Their properties, especially interfacial shear strength, can be influenced by the presence and arrangement of intermetallic compounds (IMCs) at the interface of components^3^. Moreover, morphology, size, and volume fraction of bimetal components are influential factors in determining their properties^4^. Industries are focusing on improving product quality and decreasing the manufacturing cost of bimetals^5^. Different manufacturing processes of bimetals will affect the interface characteristics of components which directly influence the properties of bimetals^6,7^. Manufacturing methods of bimetallic composites are divided into liquid–liquid, liquid–solid, and solid–solid methods based on the physical state of the base materials^8,9^. The drawbacks of liquid–liquid compound casting method are such as incomplete mixing of the two liquids, the need for complex equipment and specialized techniques, and difficulty in controlling the composition. However, the solid-state processing routes can create very good dissimilar/similar materials bonding without compromising their tensile properties^10–12^. An important method for manufacturing bimetals is compound casting, which is a liquid–solid bonding method^2,13^. The compound casting method is an easy technique to bond two metals through a liquid–solid interface, including the casting of a metallic melt around a solid metal, resulting in the joining of the two metals through a diffusion reaction^14^. Currently, Al/Cu bimetallic composites have gained significant attention in several sectors, such as automobile, electricity, and decoration fields, because of their ability to combine the unique advantages of copper and aluminum^15^. Aluminum (Al) and its alloys are known for their low cost, high conductivity, high corrosion resistance, good castability, and low density. In contrast copper (Cu) and its alloys have high electrical and thermal conductivity^1,16,17^. Moreover, they have ductile behavior due to the high symmetry of the FCC lattice in both Cu and Al. Additionally, IMCs at the Al/Cu interface can enhance both the hardness and ductility of bimetallic composites simultaneously compared to base materials by choosing appropriate manufacturing process parameters through affecting the size, morphology, and growth kinetics of the IMCs^12^. According to prior research, various IMCs may form at the Al/Cu interface under different trial conditions, such as CuAl_2_ ($\theta$), AlCu (${\eta }_{2}$), Al_3_Cu_4_ (${\zeta }_{2}$), Al_2_Cu_3_ ($\delta$), Al_4_Cu_9_ (${\gamma }_{1}$), and $\beta$ (BCC) phases^18–20^. The mechanical and physical characteristics of various IMCs impact the properties of bimetallic composites in the interfacial bond of bimetal components^21^. Cu_9_Al_4_ (${\gamma }_{1}$) exhibits the highest young’s modulus (254.69 GPa) and the lowest creep stress index of 10.75 among IMCs of Al-Cu systems, while Cu_4_Al_3_ (${\zeta }_{2}$) has the highest hardness of 11.94 GPa^20^.

Metallic lattice structures like open-cell foams with periodic properties have been widely utilized in biomedical and aerospace applications in the last decade^22,23^ due to their high stiffness-to-weight ratio, corrosion resistance, lightweight, and energy absorption characteristics^24–26^. According to the stated features, some of their applications are lightweight structures, biomedical implants, crashworthiness applications, packaging, shock absorption, buoyancy, etc.^27^. Hence, Al-based composite reinforced with copper lattice structure can have a wide range of industrial applications such as automobile, petroleum, biomedical, and aerospace^28^. Lattice structures are cellular materials composed of interconnected strut networks, nodes, and unit cells by different topologies^22^, which affect the mechanical properties of lattice structures. The uniform lattice structures with repetitive unit cells lead to uniform mechanical properties in the metallic lattice structures^24,29,30^.

In this work, we studied the appropriate casting parameters for producing Al-based bimetallic composites reinforced by a copper lattice structure to reach Al/Cu bimetallic composites with uniform properties through the compound casting of the Al/Cu bimetal. The Al liquid/Cu solid interface as a bimetal system has been assessed by a compound casting process. Al/Cu alloying at solid/liquid interface was investigated by soaking time and liquid temperature during casting and solidification time. Then, the microstructure and strength of the Al/Cu interfacial layer due to the Al-Cu diffusion band were characterized using Cu core diameters.

## Materials and methods

### Experimental method

**Figure 1:**
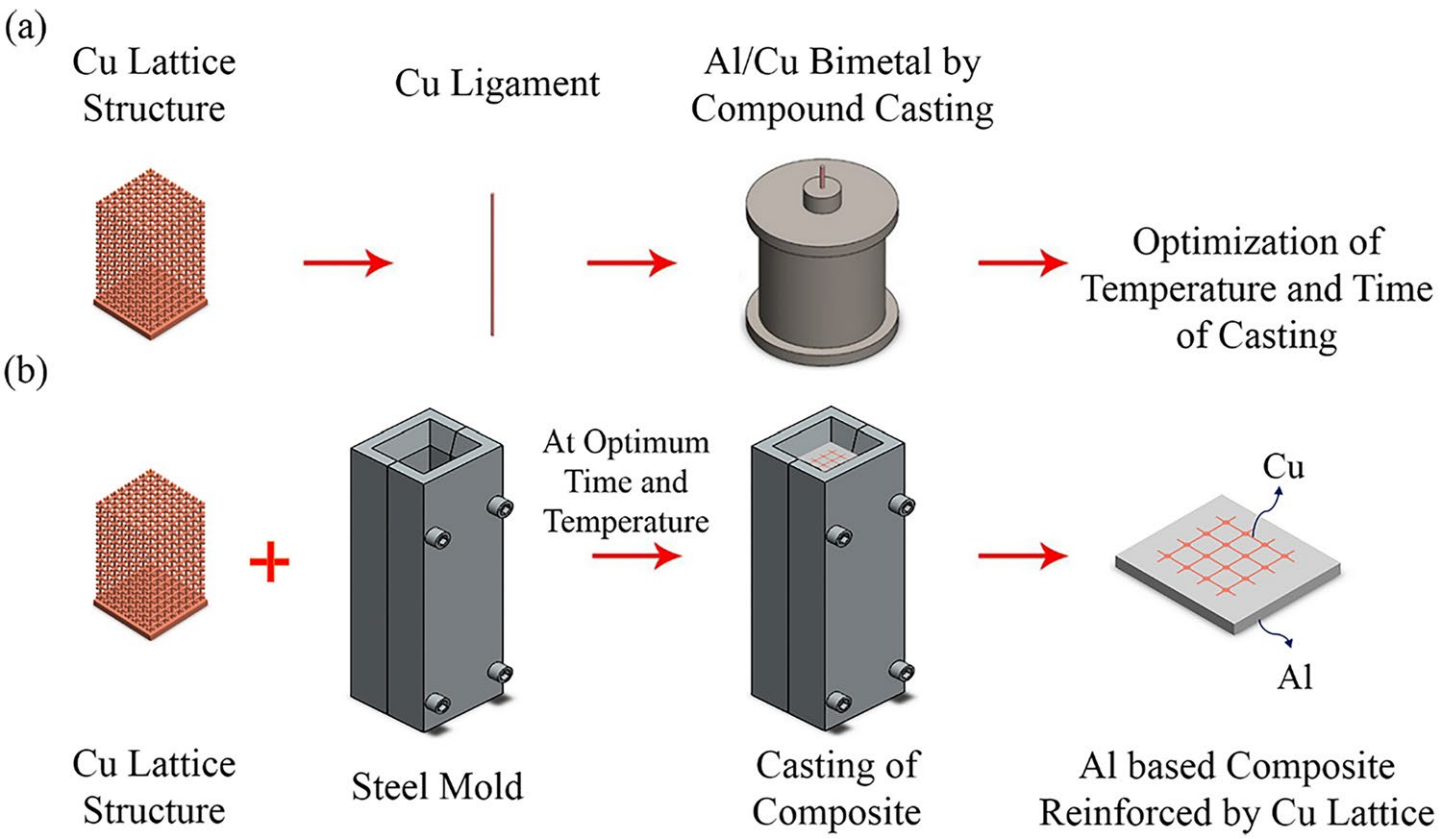
The schematic of research steps in this study, (**a**) produce Al/Cu bimetal and (**b**) produce Al based composite reinforced by Cu lattice structure.

According to Fig. 1, to manufacture the Al matrix composite reinforced by Cu lattice structure through the casting method in a steel mold, casting parameters, including temperature and time, shall be determined. Also, the optimum reduction in the diameter of the ligament of the Cu lattice structure shall be known to have appropriate diffusion bonding between the Cu lattice structure and the Al matrix. In this regard, a ligament from the Cu lattice structure as a wire was selected for optimization in this investigation. Furthermore, Cu wire was used as a Cu core in producing Al/Cu bimetal through compound casting, according to Fig. 1a. Through characterizing the Al/Cu bimetal at the Al/Cu interface, the optimum casting parameters, as well as reduction in Cu wire diameter, were determined and used in producing Al-based composite reinforced by Cu lattice structure by casting in a rectangular cube steel mold as shown in Fig. 1b. In this step, the Cu lattice structure was utilized as a reinforcement, while pure Al was selected as the matrix.

**Figure 2:**
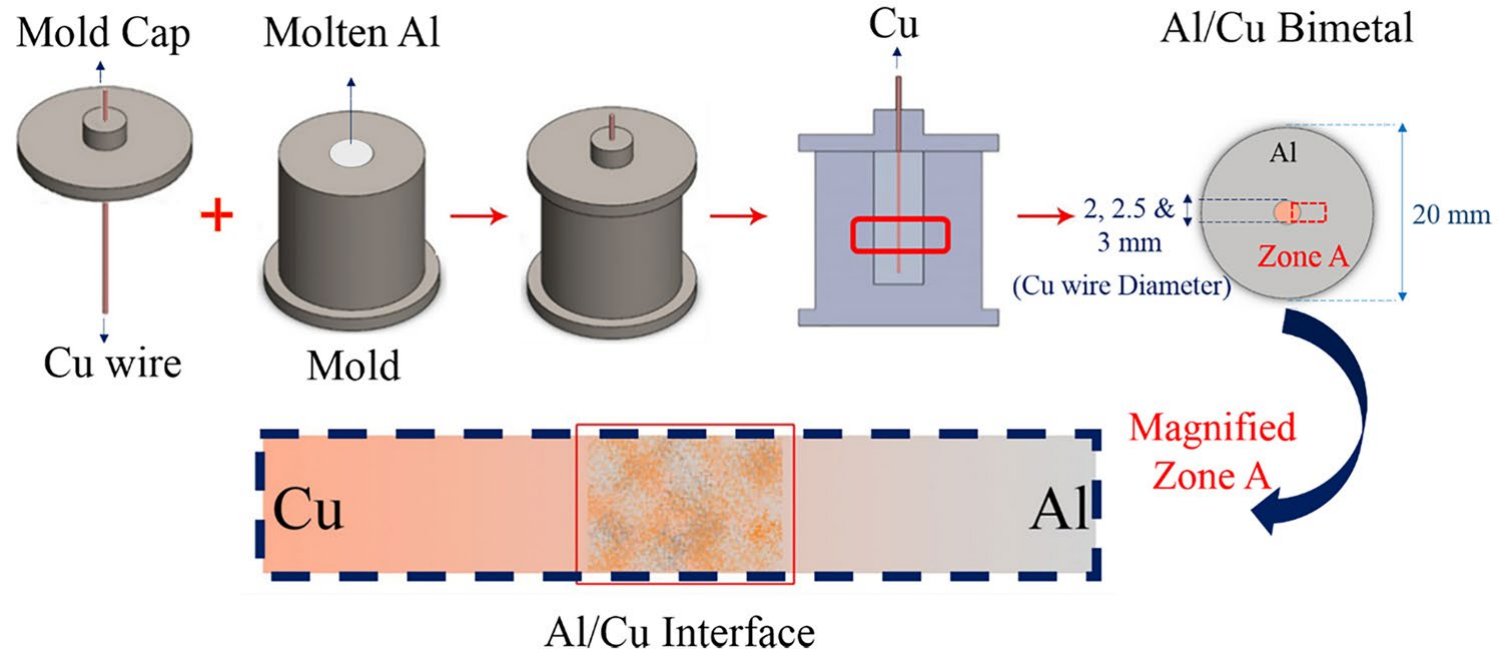
The schematic of compound casting sequences.

Figure 2 shows sequences of Al/Cu bimetal production and solid–liquid diffusion layer during compound casting. The liquid is pure Al, and solid is Cu wires with different diameters. First, pure Al ingot is melted in the induction furnace, and then molten Al is cast in a metallic mold in the electrical furnace with Ar atmosphere, which is preheated at 660 °C, and then polished Cu wire as a core is submerged at the center of the metallic mold as a bimetal set, immediately (less than 5 s). In this condition, Al liquid will be diffused in the solid Cu wire, and alloying procedure will be started at the Al(l)/Cu(s). The solid/liquid diffusion depends on the holding time at a furnace-fixed temperature. This time is named the solidification soaking time. After soaking time, the metallic mold was quenched in water, and then a piece of the cast bimetal sample was cut, according to Fig. 2, which was used to investigate the Al/Cu interface and diffusion layer.

In this study, pure copper (Cu) as wires and pure Aluminum (Al) as ingot as base materials were utilized to produce an Al/Cu bimetallic composite (Al/Cu bimetal) through the compound casting. The Cu wire with three different diameters, 2, 2.5, and 3 mm, with 60 mm in length was used as a core in bimetal specimens. Henceforth, the Al/Cu bimetal specimens consisting of Cu wires with diameters of 2, 2.5, and 3 mm will be symbolized as Al-D2Cu, Al-D2.5Cu, and Al–D3Cu, respectively. The Cu wires were cleaned before the compound casting, and the pure Al ingots were melted in a cylindrical steel mold by an electrical resistance furnace. At different mold and Al molten temperatures of 680 °C and 750 °C in an electrical resistance furnace, a Cu wire was inserted into the center of Al by using the cap of the steel mold. The temperature of the molten Al and the steel mold was kept at a constant level during the soaking period, which was 15 and 30 s. The soaking time is crucial in this work. If it is more than a certain limit, the Cu core will be dissolved completely and the Al/Cu interface will disappear. So, the effects of the soaking time on the thickness of the interface layer and properties of the Al/Cu interface. Afterward, the cylindrical steel mold was quenched in water, and finally, Al/Cu bimetal was produced. The final Al/Cu casting bimetal specimens were sliced into wafers with 5 mm thickness and were utilized for microstructural characterization and mechanical testing to assess the shear strength of the Al/Cu interface. In this study, three casting conditions are defined as 750 °C at 30 s, 680 °C at 30 s, and 680 °C at 15 s. By analyzing the bimetals, the optimum casting parameters including, mold temperature, soaking time, and Cu wire diameter, were identified, which were used to produce an Al matrix composite reinforced with Cu lattice structure. These parameters resulted in a reduction of Cu wire diameter for 10 ~ 20% through the solid solution at the Al/Cu interface of bimetal.

### Experimental tests

**Figure 3:**
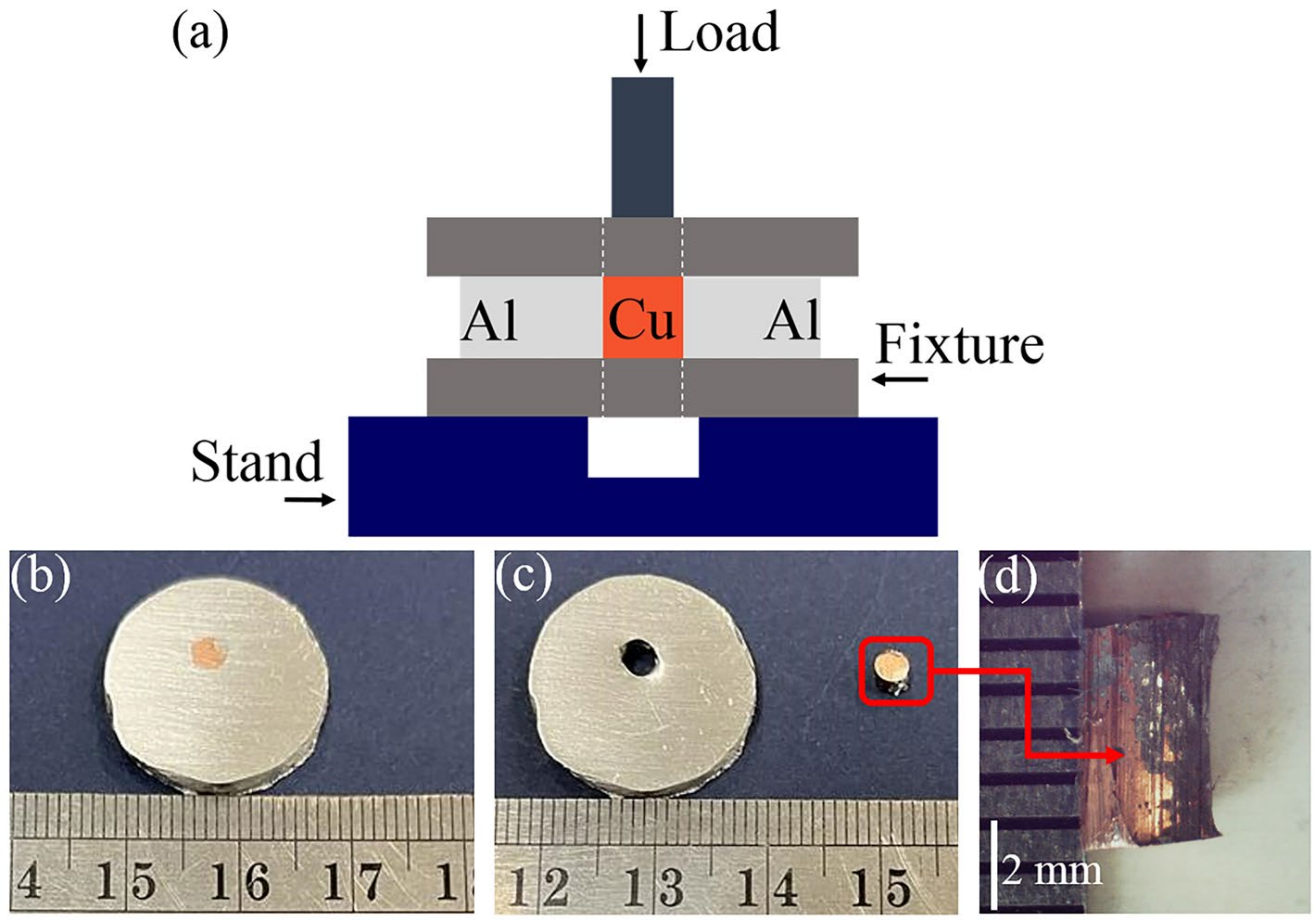
(**a**) Schematic of punch test and location of the pin on the Cu core, punch test specimen at 680 °C at 30 s (**b**) before test, (**c**) after test, and (**d**) longitudinal image from surface of Cu core after test.

To analyze the microstructure of the Al/Cu interface, bimetal specimens were observed with optical and FE-SEM microscopes with a resolution of 1.5 nm at 15 kV equipped with an energy dispersive X-ray spectrometer (EDS). To identify the phases in the specimens, X-ray diffraction equipment (XRD) was used. The mechanical properties of specimens at the Al/Cu interface were investigated by micro-hardness and punch tests. A punch test was carried out on an Instron-50 tons testing machine to study the shear strength at the Al/Cu interface of bimetals. The schematic of the utilized punch test setup is illustrated in Fig. 3a, where the tested samples were placed on a flat surface with a circular hole of 15 mm in diameter. Then, the specimens were punched using steel pins with diameters of 1.9, 2.4, and 2.9 mm. Images of punch test specimen at 680 °C at 30 s before and after test are shown in Fig. 3b and c. Also, a longitudinal image from surface of Cu core after test is shown in Fig. 3d. The displacement rate of the cross-head during punching was selected as 5 mm/min. The micro-hardness of the bimetals was examined by using the micro-hardness tester at a load of 15 g and a dwell time of 15 s across the Al/Cu interface.

## Results and discussion

The microstructural characterization of diffusion bonding, the appeared intermetallic compounds (IMCs), and mechanical characterization, including micro-hardness and shear strength, of Al/Cu bimetals at the Al/Cu interface, have been studied. As a result, the Al matrix composite reinforced by Cu lattice structure is produced using optimal parameters.

### Microstructural characterization

**Figure 4:**
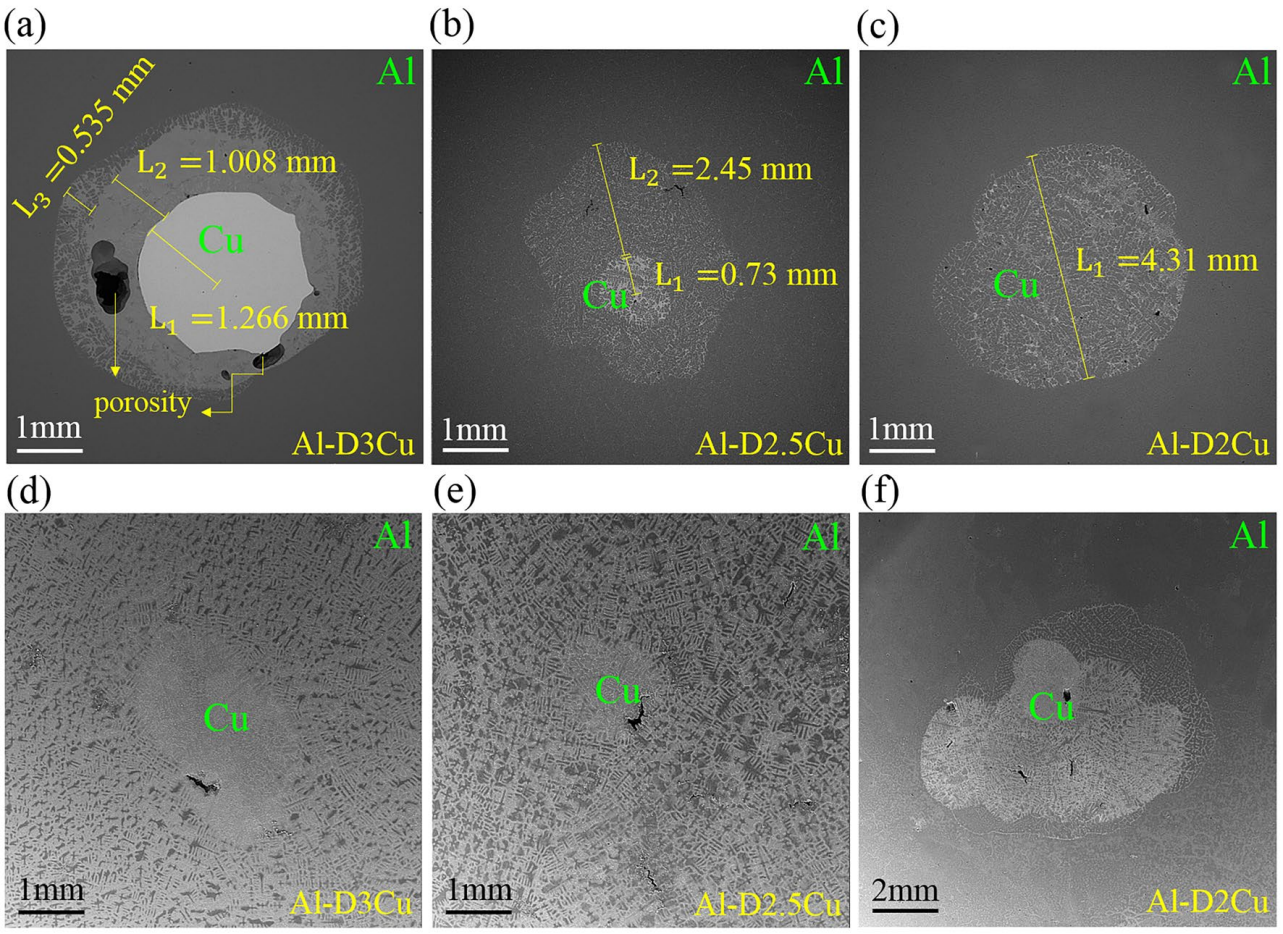
SEM images of Al/Cu bimetals at (**a**–**c**) T = 680 °C, t = 30 s and (**d**–**f**) T = 750 °C, t = 30 s.

Figure 4 shows cross-sections images of the Al/Cu bimetals produced with a casting temperature of T = 680 °C, and soaking time of t = 30 s, as well as T = 750 °C and t = 30 s. Figure 4a–c show the cross-section of Al–D3Cu, Al–D2.5Cu, and Al–D2Cu bimetals at T = 680 °C, t = 30 s, respectively. Additionally, Fig. 4d–f show the cross-section of these bimetals at T = 750 °C, t = 30 s.

As shown in Fig. 4d–f, the Cu wire cross-section was not observed in the specimens with casting condition of T = 750 °C, t = 30 s, in comparison with samples at T = 680 °C, t = 30 s, according to Fig. 4a–c. Temperature is an important factor in the diffusion process. Atoms diffuse across the interface into the reverse side if the temperature is high enough, according to the Arrhenius equation, Eq. (1), as follows^31^:

1 $$D={D}_{0}{exp}^{\left(-\frac{Q}{RT}\right)}$$

*D*, ${D}_{0}$, *Q*, *T*, and *R*, are diffusion coefficient, pre-exponential factor, activation energy, temperature of diffusion, and gas constant.

In this study, the temperature of mold and molten Al were set above the Al melting point (660 °C) at two temperatures of 680 °C and 750 °C to investigate the temperature effect on the Al/Cu interface. As revealed in Fig. 4, the Al/Cu interface became indistinct with increasing temperature, which is related to increased atom diffusion. Through the diffusion of Al and Cu atoms across the Al/Cu interface, intermetallic compounds of Al and Cu were formed in the diffusion layer. The chemical composition, name, and structure of some phases suggested by Murray^18,19^ and Liu^32^ are mentioned in Table 1. As reported by other researchers, different Al_x_Cu_y_ intermetallic compounds have different formation activation energy (E_f_), which E_f_ of some IMCs mentioned in Table 2. Regarding Table 2, CuAl_2_ has the lowest E_f_ among Al–Cu intermetallic compounds. Thus, it appears earlier than the others. Besides, the second lowest activation energy belongs to Cu_9_Al_4_. On the other hand, Cu_4_Al_3_ has the highest E_f_. Hence, it will appear rarely compared to other IMCs^33^.

**Table 1:** Chemical composition, name and structure of some intermetallic in Al–Cu system suggested by Murray^18,19^ and Liu^32^.

Stoichiometry	Symbol	Name	Lattice structure	Composition (at.% Cu)
Cu	${\alpha }_{Cu}$	Alpha	FCC	> 80.31
Cu_9_Al_4_	${\gamma }_{1}$	Gamma 1	Cubic	59.8–70
Cu_4_Al_3_	${\zeta }_{2}$	Zeta 2	Body-centered orthorhombic	55.2–56.3
CuAl	${\eta }_{2}$	Eta 2	Monoclinic	49.8–52.3
CuAl_2_	$\theta$	Theta	Body-centered Tetragonal	31.9–33
Al	${\alpha }_{Al}$	Alpha	FCC	0–2.48

**Table 2:** Formation activation energies of (*E*_*f*_) different Al_x_Cu_y_ intermetallic compounds reported in other researches.

Activation energy of formation (*E*_*f*_, eV)				Reference
CuAl	CuAl_2_	Cu_9_Al_4_	Cu_4_Al_3_	
–	0.78	0.83	–	^33,34^
–	1.01	1.23	–	^33,35^
–	–	–	1.70	^33,36^
0.93	0.74	0.88	–	^33,37^

**Figure 5:**
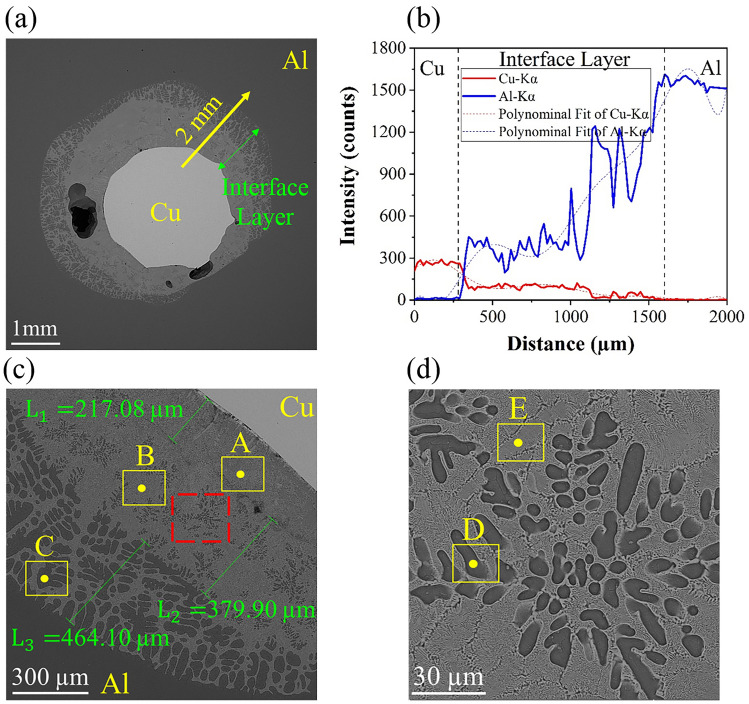
(**a**, **c**, **d**) SEM microstructural characterization, and (**b**) line scan of Al–D3Cu specimen at T = 680 °C, t = 30 s.

Pure Cu wire is only observed in the cross-section of the Al–D3Cu specimen at T = 680 °C, t = 30 s (Fig. 4a) with a decrease in the diameter of the Cu wire by about 15.6% (from 3 to 2.53 mm) due to diffusion bonding. Hence, the substructure and properties of this specimen are investigated in the following. To study Al and Cu diffusion at the Al/Cu interface of this specimen, a line scan (yellow flash in Fig. 5a) near the Al/Cu interface is shown in Fig. 5a and b. According to it, the concentration of Cu elements gradually decreases across the Al/Cu interface from the Cu side to the Al side, in contrast to the Al elements. Besides, there is no Al element at the center of the Cu wire, which shows diffusion has occurred just across the Al/Cu interface, and diffusion bonding with a specific thickness (Green flash in Fig. 5a) is observed. This phenomenon reveals that adequate interdiffusion between Al and Cu elements happens at the diffusion layer and promotes the formation of the interfacial reaction layer^38^.

Figure 5a,c,d exhibits FE-SEM images of Al–D3Cu bimetal at T = 680 °C, t = 30 s with varying magnifications. The diffusion layer, with an average thickness of 1061 µm, formed at the Al/Cu interface (Fig. 5a,c). This layer includes some porosity as shown in Fig. 4a, which is due to casting defects. This diffusion layer is composed of two main layers: layer I on the Cu side of the Al/Cu interface with a thickness of about 596.98 µm (${{\text{L}}}_{1}$+ ${{\text{L}}}_{2}$) and layer II on the Al side of the Al/Cu interface with a thickness of about 464.10 µm (${{\text{L}}}_{3}$). Both are mainly composed of two sublayers, including light and dark gray phases. The amount of dark gray phase on the Al side of the interface layer is more significant than that on the Cu side, unlike the light gray phase. Besides, in contrast to layer II, the light gray phase is the matrix phase in layer I. A magnified image of layer I (red dashed square in Fig. 5c) is shown in Fig. 5d. This layer is composed of lamellar eutectic microstructures. Considering previous research, this lamellar eutectic structure was often witnessed at the interface of Al/Cu couples^1,14,39^. To investigate the chemical composition of these layers, EDS analysis has been done at the Al/Cu interface layer, and the results of points A, B, C, D, and E (Fig. 5c,d) are mentioned in Table 3. Comparing weight percentages of Al and Cu elements of these points with other research^18,19,40^ in Table 1, it was comprehended that points A, B, and E (light gray phase) are related to CuAl_2_ ($\uptheta$), intermetallic phase of Al-Cu, since the atomic ratio of Al and Cu was 2:1. The maximum solubility limit of Cu atoms in Al is around 2.5 at.%. Regarding the low solubility of Cu atoms in Al, saturation of Cu will occur in Al solid solution^40^. Hence, points C and D (dark gray phase) are related to a combination of α-Al and CuAl_2_ ($\uptheta )$ because their EDS results exhibited that Cu atomic percent is much lower than 33.3%^32^.

**Table 3:** The EDS analysis of points in Fig. 5

Point	Al (at %)	Cu (at %)	Possible phase
A	56.46	43.55	$\uptheta$-CuAl_2_
B	62.92	37.07	$\uptheta$-CuAl_2_
C	81.43	18.57	α-Al + $\uptheta$-CuAl_2_
D	80.07	19.93	α-Al + $\uptheta$-CuAl_2_
E	67.45	32.55	$\uptheta$-CuAl_2_

**Figure 6:**
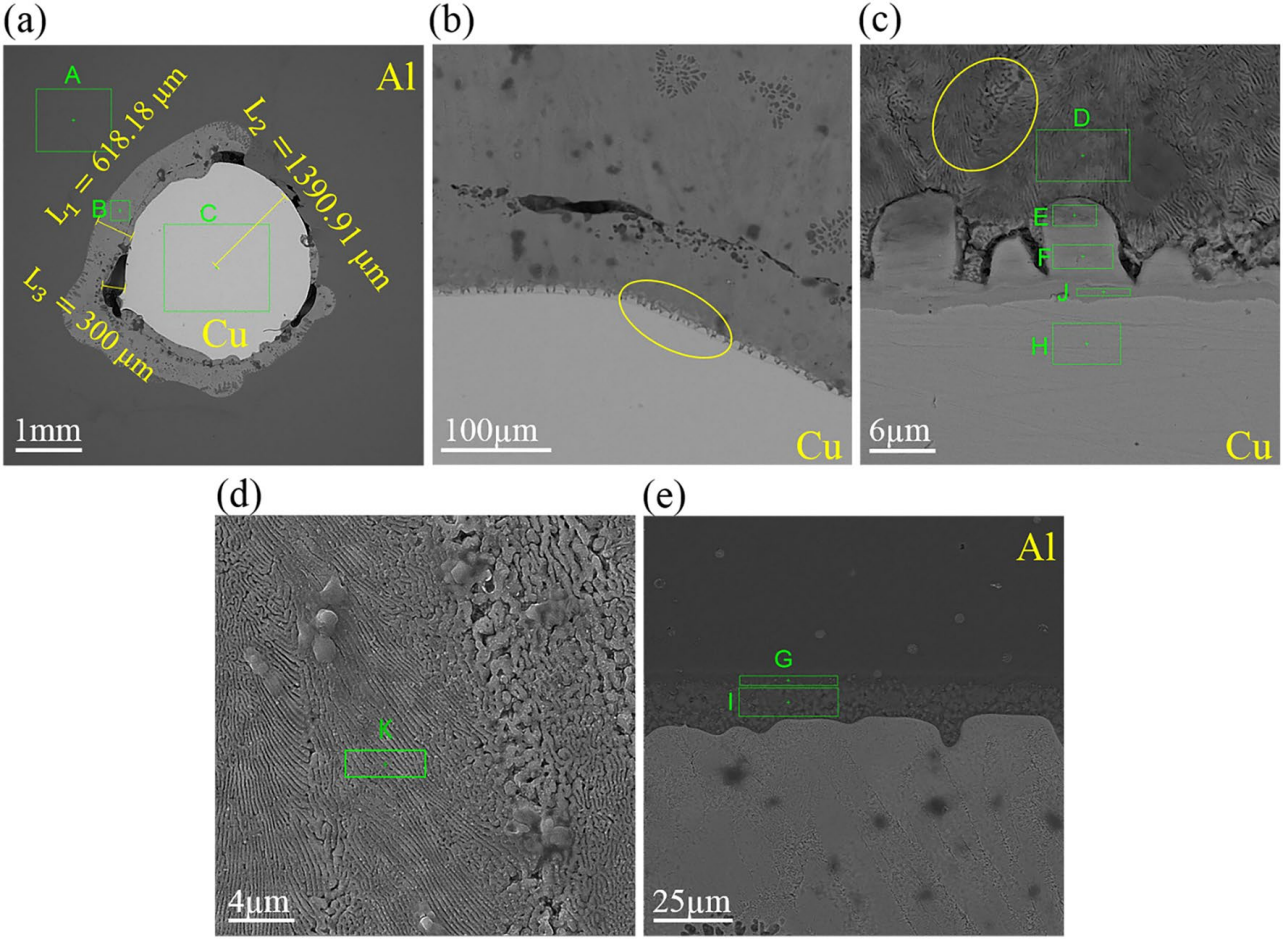
SEM image of Al–D3Cu bimetal at T = 680 °C, t = 15 s.

To disclose the diffusion layer growth at the Al/Cu interface and the effect of soaking time in the diffusion of atoms, the microstructure of the Al–D3Cu specimen at T = 680 °C & t = 15 s condition has been shown in Fig. 6. According to it, the average diffusion layer thickness of this specimen is about 618.18 µm. It is less than the diffusion layer thickness in the Al–D3Cu specimen at T = 680 °C & t = 30 s. Results show that the increase in soaking time of the casting process caused an increase in the diffusion depth of Cu and Al atoms. According to earlier research, the diffusion depth and time have a parabolic relationship, as shown in Eq. (2) ^31,41^:

2 $$d=k*{t}^{1/2}$$

where *t* is diffusion duration, *k* is the diffusion constant of solute atoms or temperature-dependent growth constant of the interface layer thickness, and *d* is the diffusion depth of solute atoms or the diffusion layer thickness. According to this equation, by doubling the time, the diffusion depth will be 1.4 times. However, according to experimental results, at a soaking time of 30 s, the diffusion layer thickness was about 1.7 times that of 15 s.

At the Al/Cu interface of the Al–D3Cu specimen at T = 680 °C & t = 15 s, some voids are seen with a maximum width of about 0.30 mm, which are related to the manufacturing process condition. The EDS analysis results of points in Fig. 6 are listed in Table 4. Comparing the EDS results with Table 1, it is understood that points H, J, and F (on the Cu side of the interface layer) are related to Cu-based solid solution considering the maximum solubility limit of Al in Cu is around 19 at.%^32^. Moreover, point E is related to the Cu_9_Al_4_ intermetallic phase. The magnified image of the interface layer is displayed in Fig. 6c and d. Figure 6d is a magnified image of the yellow oval area in Fig. 6c. Points D and k are relevant to $\uptheta$-CuAl_2_ phase, and the interface layer has a lamellar eutectic structure consisting of $\uptheta$-CuAl_2_ and α-Al + $\uptheta$-CuAl_2_ phases. Points G and I on the Al side of the interface layer are related to α-Al + $\uptheta$-CuAl_2_ and CuAl phases. As shown in Fig. 6a, the copper wire diameter decreased by about 8% (from 3 to 2.782 mm) due to solid solution and diffusion bonding, lower than the desired decrease of 10 ~ 20%.

**Table 4:** EDS analysis of points in Fig. 6

Point	Al (at.%)	Cu (at.%)	Possible phase
A	100	0	Al
B	64.58	35.42	$\uptheta$-CuAl_2_
C	0.96	99.04	Cu
D	56.34	43.66	$\uptheta$-CuAl_2_
E	35.03	64.97	Cu_9_Al_4_
F	10.76	89.24	Cu-based solid solution
G	90.66	4.83	α-Al + $\uptheta$-CuAl_2_
H	1.46	96.93	Cu-based solid solution
I	46.13	53.87	CuAl
J	18.67	81.33	Cu-based solid solution
K	68.1	31.9	$\uptheta$-CuAl_2_

**Figure 7:**
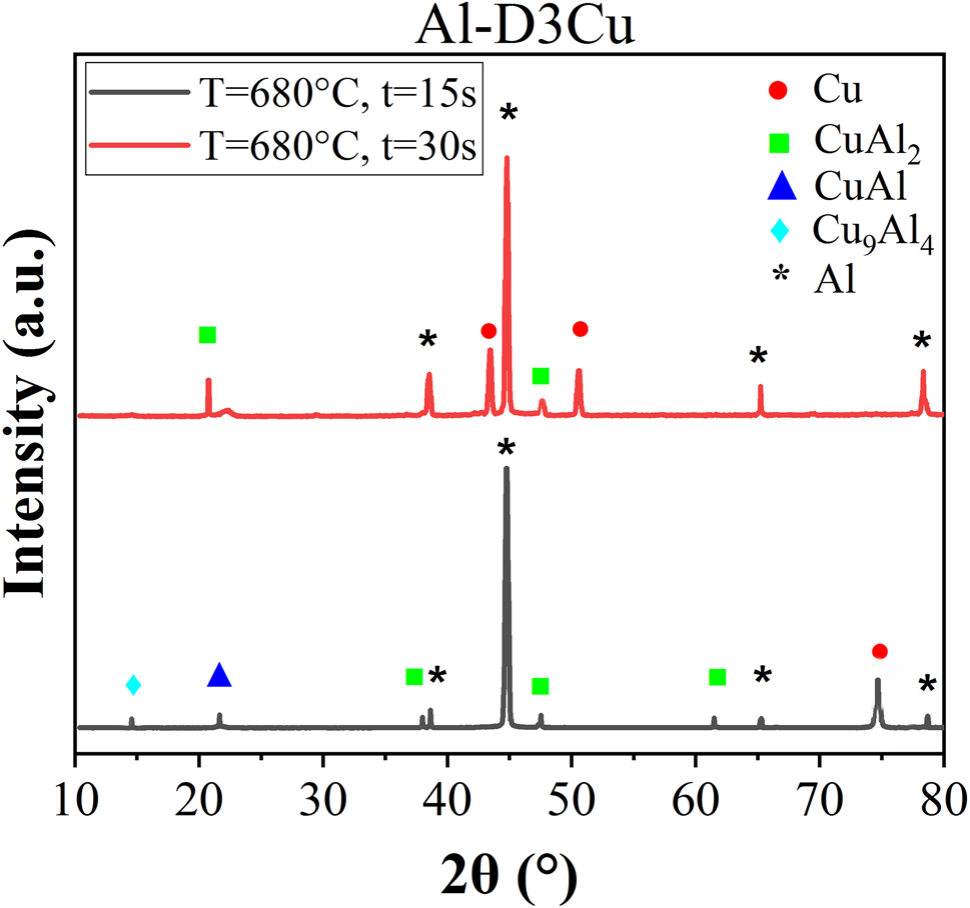
XRD results of Al–D3Cu specimen at T = 680 °C, t = 30 s, and T = 680 °C, t = 15 s.

To identify the phases in the Al–D3Cu specimens at both T = 680 °C, t = 30 s and T = 680 °C, t = 15 s conditions, and check with EDS results in Tables 3 and 4, XRD results are shown in Fig. 7. As shown, the CuAl_2_ ($\uptheta$) phase is observed as the dominant intermetallic compound at the Al/Cu interface in the Al–D3Cu bimetal at both T = 680 °C, t = 30 s, and T = 680 °C, t = 15 s.

**Figure 8:**
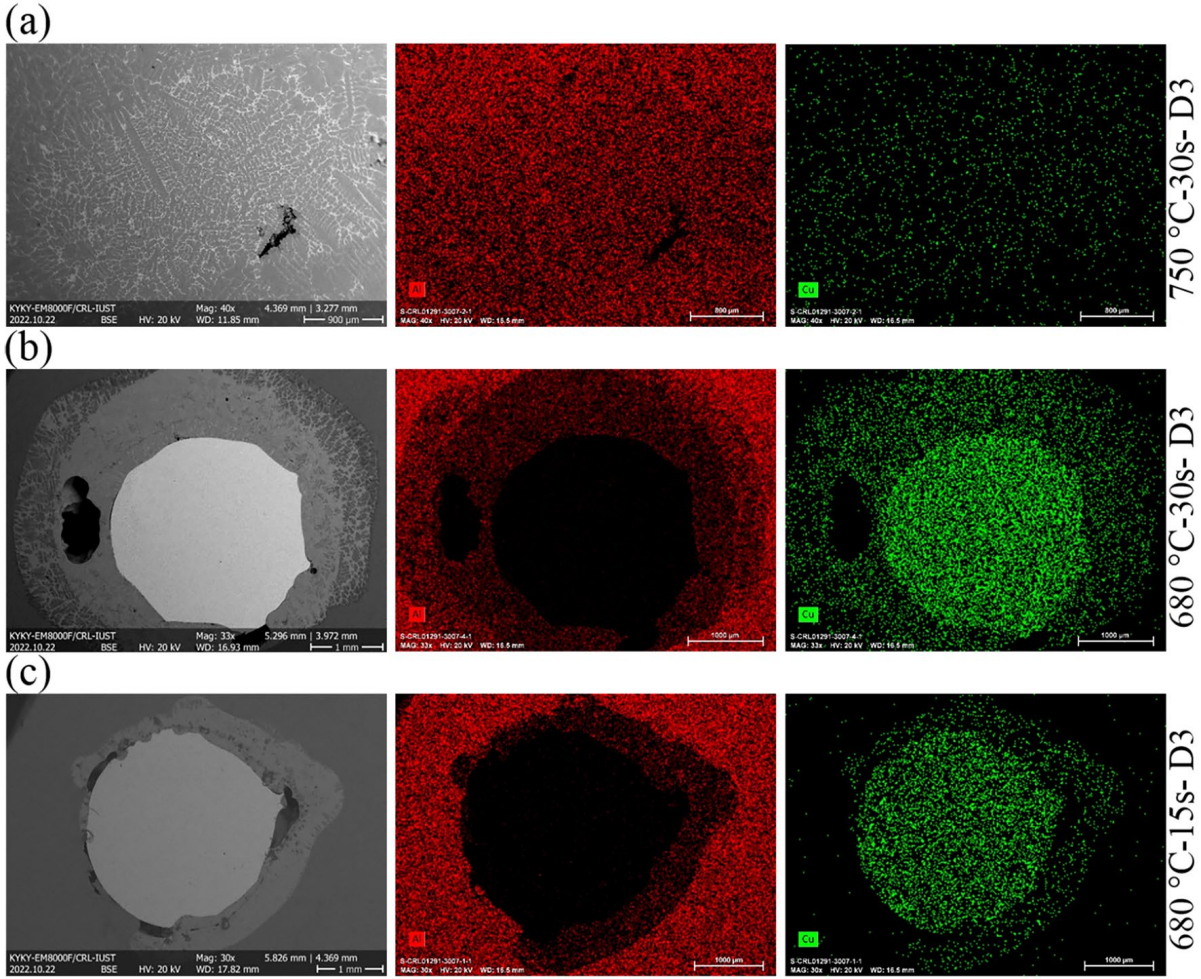
EDS maps of Al–D3Cu bimetals at (**a**) T = 750 °C, t = 30 s, (**b**) T = 680 °C, t = 30 s, and (**c**) T = 680 °C, t = 15 s.

EDS maps of Al–D3Cu specimens at three different conditions are shown in Fig. 8, with primary conditions of T = 750 °C & t = 30 s, T = 680 °C & t = 30 s, and T = 680 °C & t = 15 s to discuss the distribution of Al and Cu atoms across the Al/Cu interface in the Al/Cu bimetals. At the temperature of 680 °C (Fig. 8b,c), the atoms of Cu and Al diffused across the initial interface. Furthermore, the diffusion depth of atoms and, as a result, the thickness of the interface layer grew by increasing time from 15 to 30 s. According to former studies, the diffusion depth of Cu atoms is more sensitive to time than Al atoms. Hence, the diffusion layer growth is controlled by Cu atom diffusion in liquid Al^31^.

### Mechanical characterization

**Figure 9:**
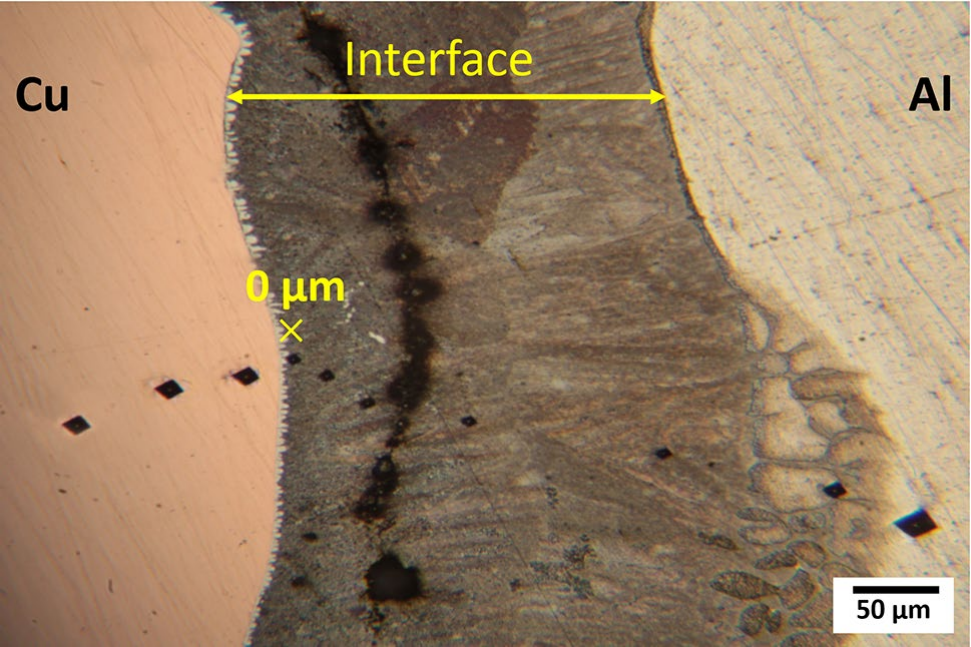
Micro-hardness indentation image of Al–D3Cu bimetal at T = 680 °C, t = 15 s.

**Figure 10:**
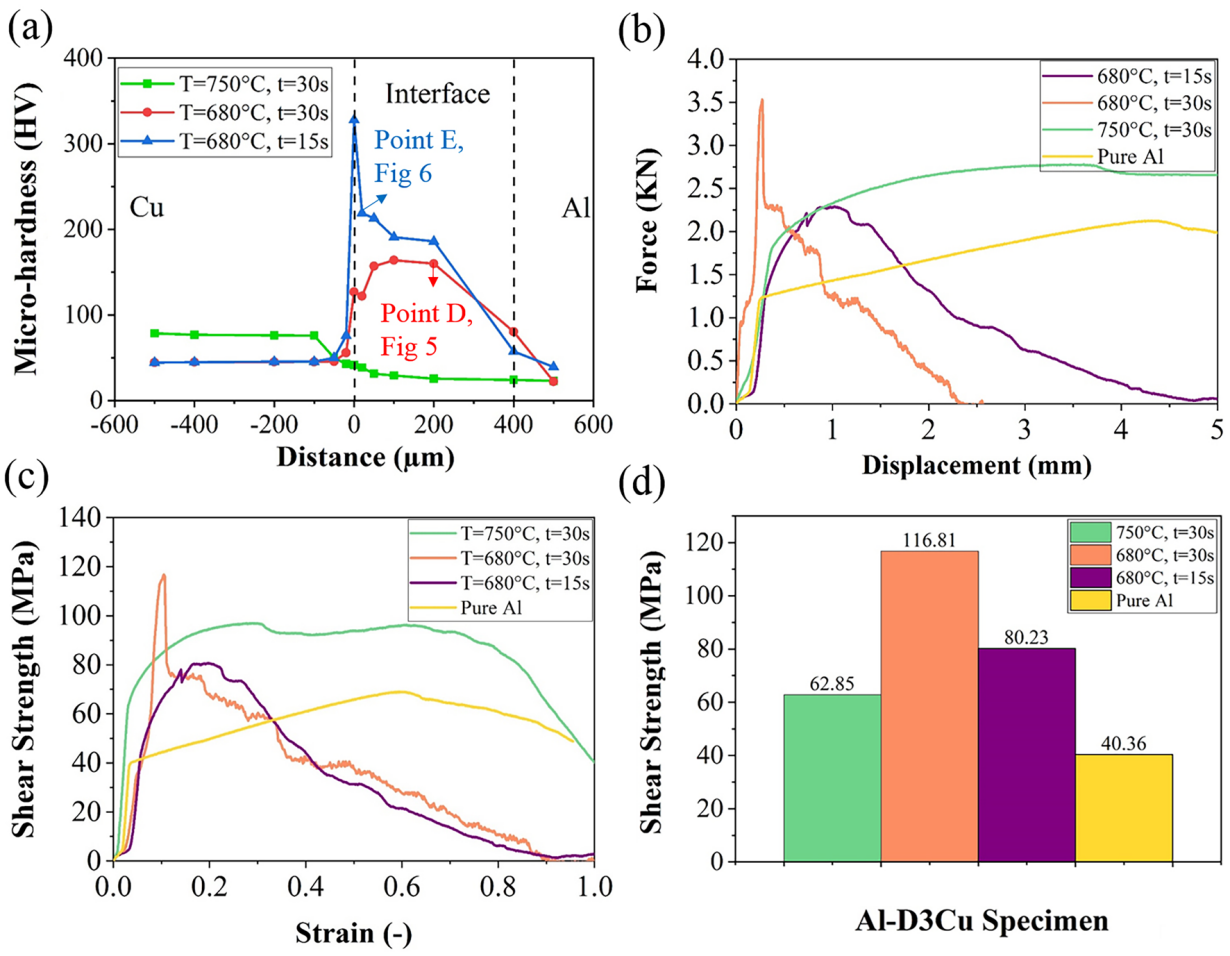
(**a**) Micro-hardness Profile of Al–D3Cu bimetals at T = 750 °C, t = 30 s, T = 680 °C, t = 30 s, and T = 680 °C, t = 15 s, (**b**) force–displacement curve, (**c**) shear strength- strain curve, and (**d**) shear strength of pure Al and Al–D3Cu bimetals at T = 750 °C, t = 30 s, T = 680 °C, t = 30 s, and T = 680 °C, t = 15 s in punch test.

As mentioned, the Al–D3Cu bimetal at T = 680 °C, t = 30 s had the optimum reduction in copper wire diameter (about 15.6%). Hence, the mechanical properties of Al–D3Cu specimens at T = 750 °C, t = 30 s, T = 680 °C, t = 30 s, and T = 680 °C, t = 15 s were characterized at the Al/Cu interface to understand the effect of temperature and soaking time on their mechanical properties. The micro-hardness (HV) of Al–D3Cu specimens has been assessed across the Al/Cu interface with distances of 20, 50, 100, 200, 400, and 500 µm on both sides of Al and Cu, as shown in Fig. 10a. Micro-hardness indentation image of Al–D3Cu bimetal at T = 680 °C, t = 15 s has been shown in Fig. 9. At T = 750 °C, t = 30 s, the Al–D3Cu specimen had the highest micro-hardness at the Cu side of the Al/Cu interface and the lowest micro-hardness at the Al side of the Al/Cu interface among all three Al/D3Cu specimens. As shown in Fig. 4d, the Al/Cu interface is not distinctive at T = 750 °C, t = 30 s and the copper core was completely dissolved during soaking time due to diffusion of atoms. In the specimens at T = 680 °C, t = 30 s, and T = 680 °C, t = 15 s, there is a sharp increase in the micro-hardness profile at the Al/Cu interface in comparison with the micro-hardness of the Cu side (around 45 HV) and the Al side (around 22 HV) of the interface, which is related to micro-hardness of Cu and Al. This is due to the formed intermetallic phases at the Al/Cu interface and observed lamellar eutectic structure, including α-Al + θ-CuAl_2_ and θ-CuAl_2_ phases (Figs. 5 and 6). The maximum micro-hardness in the Al/Cu interface of the Al–D3Cu specimen at T = 680 °C, t = 15 s (328 HV) is higher than the specimen at T = 680 °C, t = 30 s (164 HV). This is due to the observed CuAl, Cu_9_Al_4_, and CuAl_2_ intermetallic compounds at the Al/Cu interface of the Al–D3Cu specimen at T = 680 °C, t = 15 s as shown in Fig. 6 and Table 4, while only CuAl_2_ phase was observed at the Al/Cu interface of the specimen at T = 680 °C, t = 30 s (according to Fig. 5 and Table 3). These intermetallic are harder than the Aluminum base and Cu wire according to previous studies, and among them, CuAl, Cu_9_Al_4_, and CuAl_2_ have the highest micro-hardness, respectively^42^.

The diffusion layer at the Al/Cu interface and Cu core plays a considerable role in the mechanical properties of bimetals. Hence, a punch test is used to assess the bonding quality of the Al/Cu interface in the Al/Cu bimetals. The shear strength of the interface (${\tau }_{int}$) was calculated using the following Eq. (3) ^14^:

3 $${\tau }_{int}= \frac{{F}_{max}}{2\pi rl}$$

where *l* is the specimen thickness (5 mm), *r* is the insert radius, and ${F}_{max}$ is the maximum load.

Figure 10b shows force–displacement curves of punch test for pure Al and Al–D3Cu specimens at three process condition, i.e., T = 750 °C, t = 30 s, T = 680 °C, t = 30 s, and T = 680 °C, t = 15 s. Firstly, a nearly linear loading happens, and after reaching the maximum load, the load drops. The abrupt descent of the load at T = 680 °C, t = 30 s, and T = 680 °C, t = 15 s conditions reveals that the debonding of the Cu core and the Al matrix occurs^16^. Since the Cu core is completely dissolved in the Al matrix at T = 750 °C, t = 30 s due to high temperature and extended soaking time, the load's abrupt descent is not observed in this condition.

The shear strength-strain curves of pure Al and Al–D3Cu bimetals at the Al/Cu interface at different casting conditions, including T = 750 °C, t = 30 s, T = 680 °C, t = 30 s, and T = 680 °C, t = 15 s are shown in Fig. 10c. The shear strength increases with strain increment up to reaching maximum strength, then decreases without showing noticeable plastic deformation at T = 680 °C, t = 30 s, and T = 680 °C, t = 15 s conditions. However, apparent plastic deformation occurs at T = 750 °C, t = 30 s, which can be related to the complete solid solution of Cu core in the Al matrix and disappearing the Al/Cu interface in the specimen. According to Fig. 10d, the shear strength of Al–D3Cu bimetals at mentioned different casting conditions are higher than pure Al (about 40 MPa). The specimen at T = 680 °C, t = 30 s condition had the highest maximum shear strength (about 117 MPa) among the three Al–D3Cu specimens, which is related to the appeared lamellar eutectic microstructure at the Al/Cu interface including intermetallic compounds. However, the shear strength of the Al–D3Cu specimen at T = 680 °C, t = 15 s (about 80 MPa) is lower than the specimen at T = 680 °C, t = 30 s, which can be related to the lower thickness of the Al/Cu interface layer.

The area under the shear strength/strain curve of Al–D3Cu bimetals, which indicates toughness, is obtained from Fig. 10c. It can be calculated from the area under the stress–strain curve through the following Eq. (4)^43^:

4 $$W={\int }_{0}^{\varepsilon }\sigma (\varepsilon ).d\varepsilon$$

where *W*,$\varepsilon$, and $\sigma (\varepsilon )$ are strain energy, strain, and stress, separately. The highest toughness is related to the specimen at T = 750 °C, t = 30 s (about 78 ${\text{mJ}}.{{\text{mm}}}^{-3}$), and the lowest toughness is related to the specimen at T = 680 °C, t = 15 s (about 33 ${\text{mJ}}.{{\text{mm}}}^{-3}$). Also, the toughness of the specimen at T = 680 °C, t = 30 s is about 36 ${\text{mJ}}.{{\text{mm}}}^{-3}$. The difference in mechanical properties of the Al–D3Cu bimetals at different casting conditions can be related to the variation in their microstructure. Figure 4 shows that Cu wire completely dissolved in liquid Al at T = 750 °C, t = 30 s due to the diffusion of atoms at high temperature and extended soaking time, leading to solid solution formation and disappearance of the interface between Al matrix and Cu wire, as well as the lamellar eutectic structure formation. However, solid solution and lamellar eutectic structure have been formed at the interface of Cu wire and Al matrix through diffusion bonding in the Al–D3Cu bimetals at T = 680 °C, t = 30 s and T = 680 °C, t = 15 s conditions. This leads to an increase in the micro-hardness of bimetals at the Al/Cu interface through the formation of hard and brittle IMCs, causing a decrease in their toughness^42,44^.

By comparing the microstructure and mechanical properties of all Al/Cu bimetals under different casting conditions, it was found that the Al–D3Cu bimetal had the optimum reduction in Cu wire thickness at T = 680 °C and soaking time of t = 30 s, as well as a diffusion bonding with noticeable thickness at the Al/Cu interface. This interface layer improved the mechanical properties of the Al/Cu bimetallic composite. Since it is vital to partially dissolve the ligament of the lattice structure in the composite to have appropriate diffusion bonding between the ligament of the lattice structure and the Al matrix, this casting condition was selected to produce the Al matrix composite reinforced by the Cu lattice structure.

**Figure 11:**
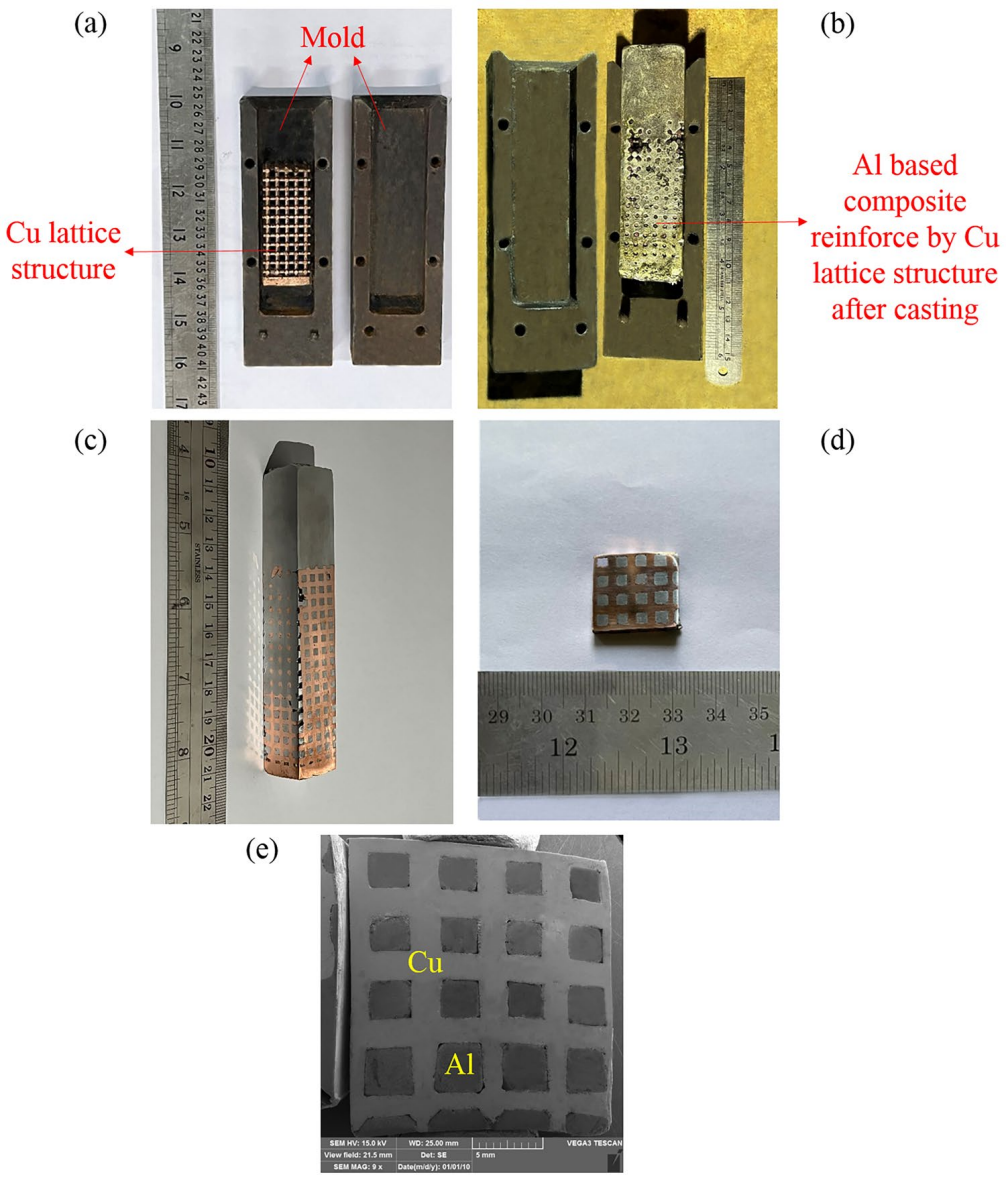
(**a**) Cu lattice structure, (**b**) Al matrix composite reinforced by Cu lattice structure after casting, (**c**) Al/Cu composite after machining, (**d**) cross-section of Al/Cu composite, and (**e**) SEM image of cross-section of Al/Cu composite.

Moreover, images of the utilized Cu lattice structure, Al matrix composite reinforced by Cu lattice structure after casting, produced Al/Cu composite after machining, a cross-section of this composite, and SEM image of cross-section of Al/Cu composite are shown in Fig. 11a–e, respectively. The microstructural and mechanical characterization of the produced Al/Cu composite will be presented in our future publication.

## Conclusion

The Al/Cu bimetallic composite (Al/Cu bimetal) was produced through the compound casting. The effect of the different temperatures and soaking times of the casting process on the microstructural and mechanical properties of the Al/Cu interface were investigated. The thickness of the Al/Cu interface and its microstructure directly affected the mechanical properties of the bimetals. The optimum casting condition, as well as a reduction in the Cu wire diameter, were experimentally obtained from the results and used to produce the Al matrix composite, which was reinforced by the Cu lattice structure. The following results can be drawn:

- The optimum reduction in the copper wire diameter by about 15.6% (from 3 to 2.532 mm) was observed in the Al/Cu bimetal with a Cu wire diameter of 3 mm (Al–D3Cu bimetal) at T = 680 °C, t = 30 s condition due to solid solution at the Al/Cu interface.
- At the Al/Cu interface with eutectic lamellar microstructure, CuAl_2_ ($\uptheta )$ phase was observed as the dominant intermetallic compound in the interface layer.
- Formed intermetallic compounds increased hardness at the interface layer. The highest hardness at the Al/Cu interface of Al–D3Cu bimetal (Al/Cu bimetal with a Cu wire diameter of 3 mm) was observed at T = 680 °C, t = 15 s condition.
- Al–D3Cu bimetals have the highest shear strength of about 117 MPa at T = 680 °C, t = 30 s condition in comparison with pure Al (about 40 MPa), while have the lowest shear strength of about 63 MPa at T = 750 °C, t = 30 s condition.
- By increasing soaking time from 15 to 30 s at the same casting temperature of 680 °C, interface layer thickness increased from 0.218 mm to 0.468 mm, which caused an increase in the shear strength from 80 to 117 MPa.
- Current work provides adequate casting processing conditions to manufacture a new Al-based composite, which is reinforced by a Cu lattice structure.

## Data Availability

The datasets generated during and/or analyzed during the current study are available from the corresponding author on reasonable request.
